# Automatic Speech Recognition in Healthcare in the Post-LLM Era: A Scoping Review

**DOI:** 10.3390/healthcare14101333

**Published:** 2026-05-13

**Authors:** Maram Alabbad, Waad Alhoshan

**Affiliations:** Computer Science Department, College of Computer and Information Sciences, Imam Mohammad Ibn Saud Islamic University (IMSIU), Riyadh 11432, Saudi Arabia

**Keywords:** Automatic Speech Recognition, large language models, healthcare, clinical documentation, scoping review

## Abstract

**Context:** Automatic Speech Recognition (ASR) in healthcare is undergoing a significant shift driven by the integration of Large Language Models (LLMs). While traditional ASR focused on transcription fidelity, LLM-based systems extend this capability to *intelligently* reason, summarize, and structure clinical data. This scoping review maps the emerging landscape of LLM-based ASR in healthcare, examining its applications, technical foundations, evaluation practices, and reported challenges. **Methods:** Following PRISMA-ScR guidelines, we searched different databases for peer-reviewed, open-access studies published between January 2022 and December 2025 to ensure reproducibility and accessibility. **Results:** Nineteen studies met the inclusion criteria from 384 screened records. Administrative documentation was the most common application (42.1%), followed by diagnosis, therapy, and doctor–patient communication. Whisper dominated ASR (52.6%), typically paired with GPT-family or LLaMA-family LLMs in frozen configurations steered through prompting. LLMs served as the primary component in 68.4% of studies. ASR evaluation within the reviewed studies predominantly relied on word error rate, while LLM evaluation remains fragmented with no standard metric. Studies reported documentation time reductions of 30–90%, though privacy reporting was inconsistent, equity concerns were rarely tested systematically, and only five studies provided replication packages. **Conclusions:** LLM-based ASR shows potential for reducing documentation burden and supporting clinical workflows, but gaps in evaluation standardization, equity testing, and reproducibility must be addressed before safe clinical deployment.

## 1. Introduction

Automatic Speech Recognition (ASR) has become increasingly important in healthcare, driven by the need to address clinician burnout, reduce documentation burden, and improve workflow efficiency. Clinical documentation consumes a substantial portion of physicians’ time, often exceeding hours spent in direct patient care, making voice-based input an attractive alternative to manual text entry [1,2,3].

ASR in healthcare has evolved through several technological generations to meet these demands. Early systems relied on Hidden Markov Models (HMMs) and required controlled acoustic environments with extensive domain-specific training [4]. The deep learning era introduced Deep Neural Networks (DNNs) for acoustic modeling, significantly improving recognition accuracy [5], while Connectionist Temporal Classification (CTC) enabled end-to-end training without frame-level alignments [6]. The Transformer architecture further revolutionized the field through efficient self-attention mechanisms [7], leading to large-scale pretrained models such as wav2vec 2.0 [8] and OpenAI’s Whisper [9]. Throughout this evolution, ASR systems remained primarily focused on transcription fidelity, measured through metrics such as Word Error Rate (WER).

Despite the advances in architectures like Whisper, recent benchmarking studies indicate that standalone ASR continues to face significant hurdles in specialized clinical environments. While ASR is increasingly adopted to alleviate documentation burdens, evaluations in neurosurgery [10] and psychiatric interviews [11] suggest that human verification remains essential due to persistent domain-specific errors. Furthermore, accuracy degrades significantly in non-English contexts without resource-intensive fine-tuning, as evidenced by recent feasibility studies in Polish medical speech [12] and French radiology [13]. These foundational challenges in acoustic modeling establish the baseline upon which generative LLMs must operate; if the raw transcription is flawed, the downstream reasoning capabilities of a stronger model may be compromised.

The emergence of Large Language Models (LLMs), such as GPT-4 [14] and LLaMA [15], has fundamentally transformed the role of ASR in healthcare [16]. Pre-trained Language Models (PLMs) like BERT [17] and its clinical variants (e.g., BioBERT [18]) serve complementary roles: while LLMs excel at generative tasks (reasoning, summarization, structured data extraction), PLMs provide discriminative capabilities for classification, entity recognition, and semantic encoding. Modern systems now extend beyond verbatim transcription to reason, summarize, and structure clinical data. This convergence of ASR and LLM technologies represents a paradigm shift—from speech-to-text conversion toward speech-to-clinical-intelligence pipelines [16]. LLM-enhanced ASR systems can generate structured clinical notes from physician-patient conversations, extract diagnostic insights from speech biomarkers, and support clinical decision-making through sophisticated natural language understanding [1,2].

Given the current transformation in the field, it is timely to examine how ASR in healthcare is evolving in the age of LLMs. This scoping review aims to map the emerging landscape of LLM-based ASR in healthcare through four angles of investigation, reporting insights and challenges. To address this aim, the following research questions—covering clinical applications, technical foundations, and evaluation practices—guide this review:

**RQ1.**  **(Applications)** In which healthcare application contexts and settings have LLM-based ASR systems been applied and evaluated?
  *Purpose: To map the landscape of healthcare applications and identify underexplored application contexts and setting areas.*
**RQ2.**  **(Technical Architectures)** What ASR and language models (including LLM and PLM), training datasets, and model adaptation techniques have been utilized in target healthcare applications?
  *Purpose: To characterize the technical diversity and identify prevailing methodological approaches.*
**RQ3.**  **(Evaluation Methods)** What evaluation environment and methods, including performance metrics, are employed to assess LLM-based ASR systems in healthcare contexts?
  *Purpose: To assess current evaluation standards and identify gaps in quality assessment practices.*
**RQ4.**  **(Reported Insights and Challenges)** What are the benefits, implementation challenges, and ethical considerations that have been reported in the included studies?
  *Purpose: To synthesize reported findings that inform deployment strategies and guide future research directions for LLM-based ASR systems intended for healthcare applications.*

To address these research questions, we conducted a scoping review following the PRISMA-ScR guidelines [19]. This methodology was selected for its suitability in mapping emerging research areas with heterogeneous methodologies and identifying knowledge gaps [20]. Given the rapidly evolving nature of LLM-based ASR in healthcare and the diversity in applications, technical approaches, and evaluation methods, a scoping review framework enables comprehensive evidence synthesis while accommodating methodological variability across studies. We limited inclusion to peer-reviewed, open-access publications to ensure accessibility, reproducibility, and alignment with open science principles. This restriction guarantees that all evidence cited in our review can be freely accessed and independently verified by researchers, clinicians, and policymakers worldwide, though it excludes subscription-journal studies and gray literature (preprints, technical reports).

This review provides the first comprehensive synthesis of LLM-based ASR in healthcare, offering: (1) a systematic mapping of applications across clinical domains; (2) an overview of technical approaches and methodological diversity; (3) a critical analysis of evaluation standards and performance metrics; and (4) evidence-based insights addressing implementation challenges, clinical benefits, and ethical considerations.

In the following sections, we present the Background and Related Work in Section 2, the Materials and Methods in Section 3, the Results in Section 4, the Discussion of Scoping Review Insights and Practical Implications in Section 5, and the Conclusion in Section 6.

## 2. Background and Related Reviews

### 2.1. Automatic Speech Recognition: Foundations and Evolution

Automatic Speech Recognition (ASR) is a core technology that enables computers to convert spoken language into written text, commonly referred to as speech-to-text (STT). In healthcare, ASR serves as a bridge for hands-free interaction with clinical systems, powering transcription of patient encounters, dictation of clinical notes, and accessibility features such as live captioning.

For decades, the industry standard for ASR relied on Hidden Markov Models (HMMs) combined with Gaussian Mixture Models (GMMs) [4]. While mathematically robust, these statistical systems were brittle and required extensive manual feature engineering. Around 2012, the integration of Deep Neural Networks (DNNs) for acoustic modeling significantly improved Word Error Rates (WER) by directly learning complex patterns from raw features [5], though they still relied on rigid HMM frameworks for sequence alignment.

The subsequent leap was to remove complex multi-component pipelines entirely using End-to-End (E2E) models. Connectionist Temporal Classification (CTC) [6] enabled networks to handle variable-length input–output sequences without requiring frame-level alignments. The Listen, Attend and Spell (LAS) architecture [21] introduced attention-based encoder–decoder models that could dynamically focus on relevant portions of the input sequence.

The introduction of the Transformer architecture [7] fundamentally changed sequence modeling through efficient self-attention mechanisms, enabling parallel processing of entire sequences rather than sequential computation. The Conformer architecture [22], proposed by Google in 2020, achieved state-of-the-art results by combining the global modeling capacity of Transformers with the local feature extraction capabilities of Convolutional Neural Networks (CNNs), achieving a WER of 2.1%/4.3% on the LibriSpeech benchmark—performance approaching human-level accuracy.

Self-supervised learning marked another transformative development. Models like wav2vec 2.0 [8] demonstrated that learning speech representations from unlabeled audio, followed by fine-tuning on transcribed speech, could outperform fully supervised approaches while requiring far less labeled data. OpenAI’s Whisper [9] leveraged large-scale weak supervision, training on 680,000 h of multilingual audio to achieve robust zero-shot performance across diverse domains, including healthcare, though concerns about hallucinations have emerged in clinical deployment contexts [23].

Throughout this evolution, ASR systems remained primarily focused on transcription fidelity, with limited capacity for semantic understanding or contextual reasoning.

### 2.2. ASR Applications in Healthcare

Before the emergence of LLMs, ASR had established a meaningful presence in healthcare across three broad areas. In clinical documentation, ASR deployments in neurosurgery [10] and radiology [13] demonstrated workflow benefits but consistently flagged accuracy limitations with domain-specific terminology, noise, and multi-speaker dialogue. In diagnostic and therapeutic assessment, ASR-based tools were developed for speech intelligibility evaluation in motor speech disorders [24] and for dysarthric speech recognition [25], revealing that general-purpose models require dedicated adaptation to serve atypical speakers reliably. Beyond transcription, ASR was applied to hearing assessment [26] and HIPAA-compliant psychiatric interview transcription [11], and comparative evaluations of end-to-end models for non-English clinical dialogue highlighted the additional challenges of real-world acoustic conditions [12].

A cross-cutting concern across these ASR-only studies is health equity. Zolnoori et al. documented significantly higher word error rates for Black patient speech compared to White patient speech in home healthcare settings [27], underscoring the risks of deploying systems trained on non-representative data in clinical environments. The same study reported a median WER of 50% for Black patients versus 33% for White patients with AWS General Transcribe (a gap of approximately 17 percentage points), and 54% versus 37% with AWS Medical Transcribe—statistically significant disparities (Brunner–Munzel test, p<0.02) indicating that the performance gap is not subtle but is large enough to carry clinical significance. Whisper, by contrast, showed no statistically significant difference between groups (72% vs. 75%, p=0.66), suggesting that model architecture and training data interact differently with dialect-related features. Comparable findings have been reported for English-language regional accents [28], and for the post-ASR correction stage, where Adedeji et al. found that LLM-based correction reduced raw WER for accented English but did not consistently close the cross-regional gap [29]. Together, these studies establish that transcription accuracy, cross-population fairness, and domain adaptation were already recognized challenges before LLM integration—challenges that the systems reviewed in subsequent sections must be understood against.

### 2.3. Large Language Models and the Convergence with ASR

The emergence of LLMs, exemplified by GPT-4 [14] and LLaMA [15], has introduced unprecedented natural language understanding and generation capabilities. Unlike earlier language models that primarily captured statistical patterns, LLMs demonstrate emergent abilities including complex reasoning, instruction following, and in-context learning [30]. In healthcare, LLMs have shown remarkable potential across clinical decision support, medical education, patient communication, and administrative tasks [16,31]. A systematic review documented a dramatic surge in research interest, with publications increasing from a single study in 2019 to over 550 by 2024 [32]. However, deployment has revealed significant limitations, including variable performance across medical specialties and hallucinations—confident generation of factually incorrect information—which remain critical concerns in high-stakes clinical contexts [33,34].

The integration of ASR and LLM technologies suggests an early transition from speech-to-text conversion toward speech-to-clinical-intelligence pipelines. In this emerging model, spoken clinical encounters are not merely transcribed but processed, enriched, summarized, and mapped to clinical concepts through the contextual understanding embodied in LLMs.

A recent study of an “AI scribe” in plastic and reconstructive surgery demonstrated how LLM-guided transcription can reshape clinical documentation workflows [3]. Similar systems are being deployed across diverse settings, with companies integrating LLM capabilities into ambient clinical documentation tools. However, this rapid deployment has outpaced rigorous evaluation, raising concerns about accuracy, safety, and appropriate use [23]. The complexity of multi-component systems introduces new failure modes, including cascading errors where ASR mistakes are amplified by LLM processing, and hallucinations that may introduce clinically dangerous misinformation into medical records.

**Figure 1:**
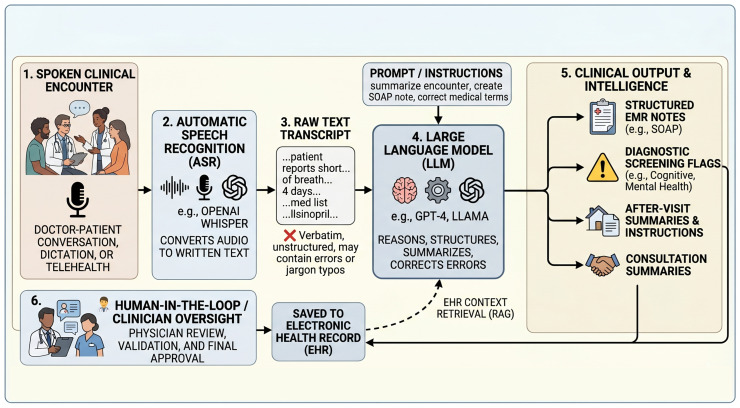
Schematic of a typical ASR–LLM clinical pipeline.

Figure 1 provides a schematic of a typical ASR–LLM clinical pipeline, summarizing the common stages that recur across the included studies and clarifying the points at which errors can propagate and at which human oversight is naturally integrated.

### 2.4. Related Reviews and Research Gaps

Several recent reviews have examined specific aspects of speech technology and language models in healthcare. However, none have comprehensively addressed the rapidly emerging integration of ASR with LLMs. Table 1 provides a systematic comparison of related reviews, highlighting the unique contribution of this work.

Van Buchem et al. (2021) conducted an early scoping review on digital scribes in clinical practice [35], establishing a foundational understanding of ASR-based documentation systems, but predates the emergence of LLMs and focuses on traditional NLP pipelines. Sasseville et al. (2025) systematically reviewed AI scribes for clinical documentation [37], examining their impact on workflow efficiency and documentation quality, but did not specifically address LLM integration or provide detailed technical architecture analysis. Ng et al. (2025) conducted a systematic review on ASR performance for clinical documentation [2], but focused primarily on traditional metrics like Word Error Rate without systematic examination of LLM integration. Jordan et al. (2025) examined Speech Emotion Recognition for mental health [36], a specialized sub-field that analyzes emotional states from voice characteristics rather than transcription accuracy. Zhang et al. (2023) surveyed intelligent speech technologies across transcription, diagnosis, and equipment control [1], but predates the significant advancements in generative AI reshaping the field.

In the LLM domain, multiple reviews have examined healthcare applications [16,30,34], but have not specifically addressed convergence with speech recognition technology.

Beyond healthcare-focused reviews, recent surveys have examined ASR-LLM integration in general domains (Table 2). Muthusamy et al. (2025) provided a comprehensive overview of LLM impact on ASR systems across 60+ years of research, focusing on general-domain applications in English and Indian languages [38]. Yang et al. (2025) surveyed technical integration approaches when large language models meet speech, examining fusion methods across domains [39]. Cui et al. (2025) reviewed recent advances in speech language models, covering architectural innovations and training paradigms [40]. While these works establish the technical landscape of ASR-LLM integration, they do not address healthcare-specific challenges such as clinical safety validation (cascading error mitigation, human-in-the-loop verification), medical terminology handling, regulatory positioning (FDA SaMD, HIPAA/GDPR compliance), equity evaluation (subgroup fairness testing), or reproducibility requirements for clinical deployment.

This review addresses these gaps by focusing explicitly on the convergence of ASR and LLMs in healthcare. We extend beyond traditional accuracy metrics to examine semantic and reasoning capabilities, encompass the full spectrum of healthcare domains, and focus specifically on the transformative post-LLM era. By synthesizing evidence on clinical applications, technical architectures, evaluation methodologies, and ethical considerations, this work offers a comprehensive roadmap for LLM-enhanced speech recognition in healthcare.

## 3. Review  Methodology

This scoping review adheres to the methodological framework established by Arksey and O’Malley [41] and follows the reporting guidelines outlined in the PRISMA Extension for Scoping Reviews (PRISMA-ScR) [19].

**Protocol Registration.** This scoping review protocol was registered with the International Platform of Registered Systematic Review and Meta-analysis Protocols (INPLASY) on 10 April 2026, registration number INPLASY202640033 (DOI: 10.37766/inplasy2026.4.0033; available at: https://inplasy.com/?s=INPLASY202640033) (accessed on 11 April 2026). There were no deviations from the registered protocol during the conduct of this review.

### 3.1. Eligibility Criteria

Studies were selected based on predefined inclusion and exclusion criteria in accordance with PRISMA-ScR guidelines. To be considered eligible, studies had to meet the following criteria:

- Published between 1 January 2022 and 31 December 2025.
- Original research articles (empirical, experimental, or solution-based studies).
- Full text available in English and open-access.
- Focus on ASR in healthcare or health-related contexts (e.g., clinical documentation, diagnosis, therapy, patient communication, medical education, accessibility, administration).
- Investigation of integrated ASR-LLM pipelines for downstream clinical tasks.

Studies were excluded if they: (1) lacked an ASR component, (2) did not integrate LLM capabilities, (3) focused on non-clinical or non-health applications, or (4) were secondary research such as reviews or surveys.

### 3.2. Information Sources and Search Strategy

The search encompassed four prominent scientific databases: PubMed, Scopus, IEEE Xplore, and Web of Science. These databases were selected to ensure comprehensive coverage of biomedical, engineering, and computer science literature relevant to ASR and LLM applications in healthcare. The review covered literature published from January 2022 through December 2025, capturing developments following the release and widespread adoption of large language models.

The search query was constructed from three primary components combined using the AND operator: (1) Automatic Speech Recognition terminology, including “automatic speech recognition”, “speech recognition”, “speech-to-text”, and “voice recognition”; (2) healthcare domain terms, including “healthcare”, “medicine”, “clinical”, “patient”, “hospital”, and “medical”; and (3) Large Language Model identifiers, including “Large Language Model”, “LLMs”, “GPT”, and “Whisper”. The search strategy was developed through a systematic and iterative process, beginning with an initial set of terms informed by existing literature, which were progressively refined based on relevance and precision. A validation set of five relevant studies was used to assess the sensitivity and specificity of the search string, ensuring adequate capture of relevant articles while minimizing irrelevant results. The  exact database queries with field tags and date filters, along with the five validation studies, are reproduced in Appendix B.

### 3.3. Selection of Sources of Evidence

**Figure 2:**
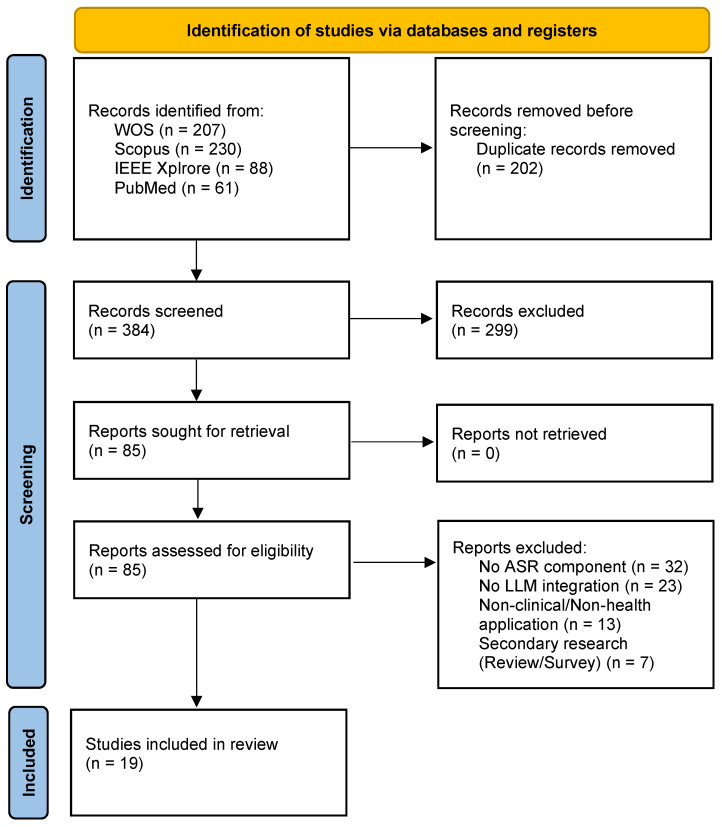
PRISMA flow diagram illustrating the study selection process.

The study selection process followed the PRISMA 2020 guidelines and involved three phases: identification, screening, and inclusion. Figure 2 illustrates the complete selection process. The initial search across all four databases yielded 586 records (Web of Science: 207; Scopus: 230; IEEE Xplore: 88; PubMed: 61). Given the overlapping indexing across Web of Science, Scopus, IEEE Xplore, and PubMed, a significant number of identical records were retrieved. Deduplication was performed systematically using a citation management tool, which matched records based on title, authors, publication year, and DOI. All automated matches were subsequently verified manually by the reviewers to ensure no unique studies with similar titles were inadvertently excluded. After removing 202 duplicate records, 384 unique records remained for screening. Title and abstract screening excluded 299 records that did not meet the eligibility criteria. The remaining 85 reports were retrieved for full-text assessment, all of which were successfully obtained. Full-text review resulted in the exclusion of 57 studies for the following reasons: no ASR component (n = 32), no LLM integration (n = 23), non-clinical or non-health application (n = 13), and secondary research (n = 7). This process yielded a final sample of 19 studies included in the review.

### 3.4. Data Charting Process

A structured data extraction form was developed to systematically extract relevant information from the 19 included studies. To ensure consistency, the form was piloted on an initial subset of studies and subsequently refined. Two reviewers independently extracted the data, with discrepancies resolved through discussion and consensus. The extraction scheme, presented in Table 3, maps each data item to its corresponding research question(s). The extracted data was organized into seven key domains:

1. **Bibliographic Metadata:** Author(s), country, publication year, source, and study type.
2. **Study Context:** Research motivation, objectives, application context, clinical setting, target population, and supported languages.
3. **Technical Architecture:** LLMs employed, ASR system specifications, dataset characteristics, and adaptation techniques.
4. **Evaluation and Validation:** Methodologies, performance metrics, benchmark datasets, and human-in-the-loop requirements.
5. **Clinical Outcomes:** Time savings, documentation quality, and user satisfaction.
6. **Ethics and Implementation:** Privacy, bias considerations, and regulatory compliance.
7. **Reproducibility:** Availability of code, models, and datasets.

**Table 3:** Data extraction scheme.

Extraction Domain	Item to Extract	Corresponding RQ(s)
Bibliographic Metadata	Author(s), first author’s country, publication year, source, paper type	N/A
Study Context	Application context (e.g., diagnosis, admin)	RQ1
	Setting (e.g., hospital, telehealth)	RQ1
	Target population (e.g., clinicians, patients)	RQ1
	Language(s) supported	RQ1
	Paper motivation (intended health problem)	RQ1
Technology and Methods	ASR model(s) used (e.g., Whisper, Google STT)	RQ2
	LLM(s) used (e.g., GPT-4, LLaMA)	RQ2
	Dataset(s) used (nature, size, availability, modality)	RQ2
	Adaptation technique (e.g., fine-tuning, prompting)	RQ2
	System features (input/output; open-source/proprietary)	RQ2
Evaluation and Validation	Validation methods and metrics (e.g., WER, accuracy)	RQ3
	External evaluation methods (e.g., user studies, clinical simulation)	RQ3
	User involvement in testing	RQ3
	Human-in-the-loop required	RQ3
Outcomes Beyond Accuracy	Clinical utility (time saved, satisfaction, workload)	RQ3, RQ4
Ethics and Implementation	Privacy and data governance measures	RQ4
	Equity considerations (accents, low-resource languages)	RQ4
	Adoption factors (integration, cost, barriers)	RQ4
Replication	Availability of replication package (code, data, models)	RQ4

## 4. Results

### 4.1. Study Characteristics

**Figure 3:**
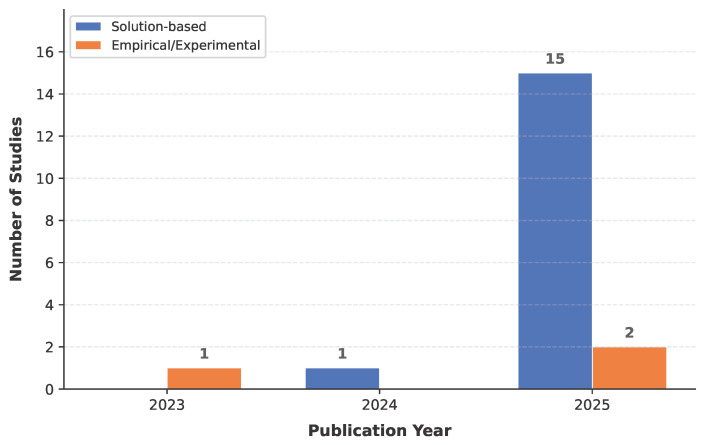
Distribution of included studies by publication year and study type (N = 19). Solution-based studies propose novel systems or pipelines, while empirical studies evaluate existing tools.

A total of 19 studies met the inclusion criteria, all published as peer-reviewed journal articles between 2023 and 2025. The studies were retrieved from several academic databases, including Web of Science, Scopus, IEEE Xplore, and PubMed. Figure 3 shows the distribution of studies by publication year and study type.

Publications were sparse before 2025, with one study in 2023 [42] and one in 2024 [43], before surging to 17 in 2025 and accounting for 89.5% of the included studies. This growth is consistent with the broader expansion of LLM research in healthcare [16] and the increasing availability of open-source speech models during this period [44]. The sharp surge of publications in 2025 directly reflects the clinical research lifecycle. Following the widespread release of foundational tools like ChatGPT (late 2022) and the Whisper API (2023), researchers required time for IRB approvals, data collection, and peer review, resulting in a natural clustering of published literature in 2025.

Regarding study type, 16 studies (84.2%) were solution-based, proposing new systems, pipelines, or frameworks that integrate ASR with LLM components for clinical tasks. The remaining three (15.8%) were empirical, focusing on evaluating or benchmarking existing tools [42,45,46].

**Figure 4:**
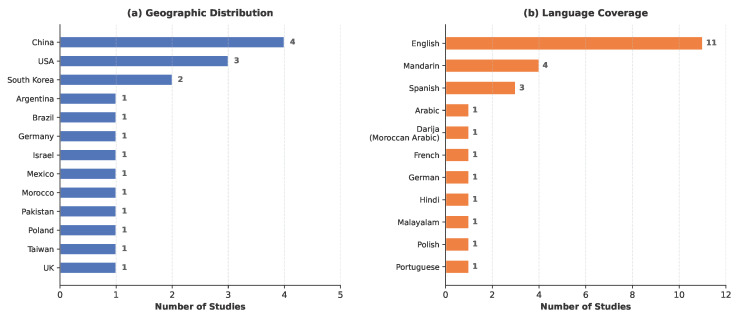
Distribution of included studies by (**a**) first-author country of affiliation and (**b**) supported language(s). A single study may support multiple languages; therefore, language counts exceed N = 19.

Based on first-author affiliation, the included studies spanned 13 countries across Asia, Europe, the Americas, and Africa, reflecting broad geographic interest in integrating LLMs with ASR for healthcare. China contributed the largest share (n = 4), followed by the USA (n = 3) and South Korea (n = 2), with the remaining studies distributed across single-country contributions from Poland, Israel, Taiwan, Argentina, Brazil, Morocco, Germany, Pakistan, Mexico, and the UK. Linguistically, the studies collectively addressed 11 languages (Figure 4). English was the most frequently targeted language, appearing in 11 studies (57.9%), followed by Mandarin (n = 4) and Spanish (n = 3). Seven studies (36.8%) operated exclusively in English, while eight (42.1%) targeted non-English languages only—including Mandarin, Polish, Portuguese, and Spanish—and four (21.1%) adopted multilingual configurations supporting two or more languages [47,48,49,50]. Notably, one study addressed Darija—a spoken Arabic dialect of Morocco—with French code-switching [51], highlighting emerging efforts toward low-resource and dialectal language support in clinical ASR.

Overall, the higher proportion of solution-based work suggests that much of the current research effort is directed toward building and demonstrating new approaches, while empirical evaluation of these tools in clinical practice is still emerging. Moreover, although the geographic spread is encouraging, the concentration of studies on English and a limited number of high-resource languages underscores a gap in coverage for underrepresented languages and dialects. The following subsections examine this evolving landscape in greater depth, presenting findings across the four research questions to reveal how these systems are being applied (RQ1), what technical foundations they rely on (RQ2), how they are evaluated (RQ3), and what challenges and insights have been reported (RQ4).

### 4.2. RQ1: Clinical Applications and Healthcare Domains

This subsection answers the first research question: *In which healthcare application contexts and settings have LLM-based ASR systems been applied and evaluated?* We examined four dimensions across the 19 included studies: application context, clinical setting, language–application context and setting interaction, and target population reported in the included studies.

Four application contexts were identified, as defined in Table 4. Administrative documentation was the most common (n = 8), driven by the widely cited need to reduce clinician documentation burden, EHR fatigue, and burnout [47,51,52,53,54]. Diagnostic applications (n = 4) covered a range of clinical areas, from Alzheimer’s detection [43,55] to stress detection [56] and automated therapy session coding [45]. Therapy-oriented studies (n = 4) were motivated by the growing demand for accessible mental health services [57,58] and explored VR-based counseling [58], therapeutic dialogue systems [42], teletherapy augmentation [57], and bilingual patient education [48]. Doctor–patient communication studies (n = 3) addressed challenges such as emergency speech reconstruction [50], dysarthric speech recognition [59], EMR generation from consultations [60], and surgical note generation [61]. Across these contexts, two additional motivational threads emerged: addressing speech and communication disorders [56,59] and advancing health equity through multilingual support [48,50,51].

**Figure 5:**
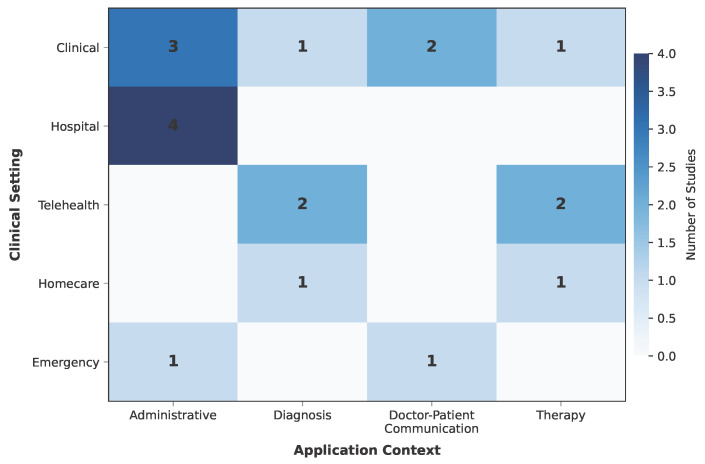
Heatmap of application context mapped against clinical setting (n = 19). Color intensity indicates study count. Administrative applications are the most broadly distributed across settings, while diagnostic and therapeutic studies cluster in telehealth and homecare environments.

Figure 5 presents a heatmap mapping these application contexts against five clinical settings, defined in Table 5. Clinical environments (outpatient clinics, specialty practices) were the most represented (n = 7), followed by hospital settings (n = 4) and telehealth platforms (n = 4), with homecare (n = 2) and emergency (n = 2) settings less frequently targeted. This distribution suggests that a substantial portion of research is already targeting real or near-real clinical environments rather than remaining confined to controlled laboratory conditions. As shown in Figure 5, administrative applications were the most broadly distributed, spanning clinical (n = 3), hospital (n = 4), and emergency (n = 1) environments. Hospital-based administrative work accounted for the highest single-cell concentration, driven by radiology transcription [49], ophthalmic documentation [47], orthodontic records [46], and tumor board compliance [51]. In contrast, diagnostic and therapeutic studies clustered in telehealth and homecare settings, reflecting the remote and patient-initiated nature of these applications. Emergency settings accounted for only two studies [50,54], pointing to a gap in high-acuity deployment despite the potential value of real-time ASR-LLM support in time-critical scenarios.

**Figure 6:**
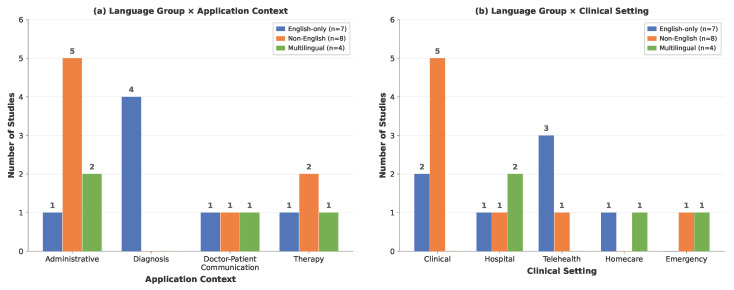
Distribution of studies by language group across (**a**) application context and (**b**) clinical setting (n = 19). English-only studies concentrate on diagnosis in telehealth settings, while non-English studies focus on administrative and communication tasks in clinical environments.

The interaction between language group, application context, and clinical setting reveals distinct research profiles, as illustrated in Figure 6. English-only studies (n = 7) concentrated heavily on diagnosis (4 out of 7) and were most often situated in telehealth settings (n = 3), consistent with a research emphasis on proof-of-concept systems for speech-based screening and detection. Non-English studies (n = 8) exhibited the opposite profile: they were predominantly oriented toward administrative tasks and doctor–patient communication (6 out of 8), and five out of eight were conducted in clinical environments [42,52,59,60,61]. pattern suggests that non-English research is more often motivated by immediate clinical workflow needs—such as documenting consultations in Portuguese, Polish, Spanish, or Mandarin—rather than by technical benchmarking. This divergence likely stems from a fundamental infrastructural gap: while English-language researchers can leverage mature, commercially available medical ASR tools to experiment with advanced diagnostic applications in telehealth, non-English environments often lack robust baseline models. Consequently, non-English research is compelled to focus on solving foundational hurdles within local clinical settings. Multilingual studies (n = 4) occupied a middle ground, spanning administrative (n = 2), therapy (n = 1), and doctor–patient communication (n = 1) contexts, and were deployed in hospital (n = 2), homecare (n = 1), and emergency (n = 1) settings, reflecting the more diverse deployment demands of systems designed for multiple language communities.

Regarding the target population in the 19 included studies, clinicians were the most frequently targeted (n = 12), particularly in administrative and doctor–patient communication applications. Patients were targeted in nine studies, predominantly in diagnostic and therapeutic contexts. Three studies explicitly served both populations [45,57,60], and one study targeted administrative personnel [50].


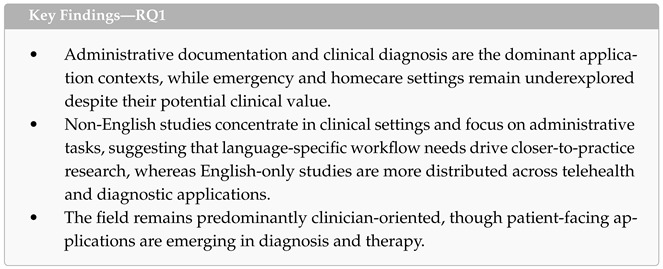


### 4.3. RQ2: Technical Architectures

This subsection addresses the second research question: *What models (including LLM and PLM), training datasets, and model adaptation techniques have been utilized in target healthcare applications?* We analyzed the technical architecture of all 19 included studies across three pipeline components—ASR systems, generative LLMs, and supporting pre-trained language models (PLMs)—followed by a cross-cutting analysis of adaptation patterns across the ASR-LLM pipeline.

**Figure 7:**
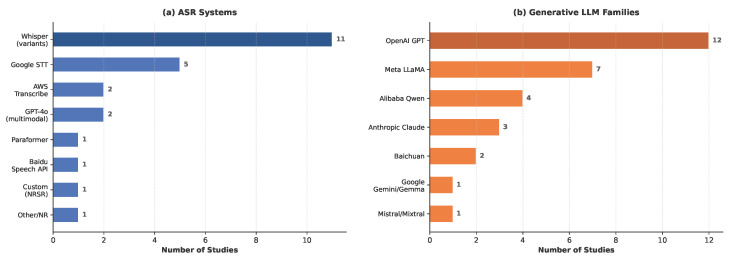
Distribution of (**a**) ASR systems and (**b**) generative LLM families across the 19 included studies. A single study may employ multiple ASR systems or LLMs; therefore, counts may exceed n = 19. Whisper and OpenAI GPT are the dominant choices for ASR and LLM components, respectively.

**ASR Component.** OpenAI’s Whisper was the dominant ASR system, employed in 11 of the 19 studies across multiple model sizes, including Large [43,51,55], Medium [53,59], and Small [53], as shown in Figure 7a. Four studies used Whisper without specifying the variant [45,48,52,61]. Cloud-based commercial APIs formed the second tier, with Google Speech-to-Text appearing in five studies [42,45,46,50,58] and AWS Transcribe in two [46,57]. A notable emerging trend was the use of GPT-4o as a unified ASR-LLM system, serving dual transcription and language understanding roles in two studies [46,49]. Only two studies employed non-mainstream ASR solutions: a custom noise-robust model (NRSR/DeNoiseformer) designed for ambulance environments [54] and Paraformer for Chinese medical speech [47].

ASR served a secondary role in the majority of studies (12 out of 19), functioning as a transcription front-end that feeds into downstream LLM processing. In the remaining seven studies, ASR was the primary contribution, either because the study focused on ASR performance itself [46,53] or because domain-specific ASR adaptation was a core technical contribution [47,49,51,54,59].

The vast majority of studies (15 out of 19) used pre-trained ASR models in a frozen configuration without any domain-specific adaptation. Only four studies fine-tuned their ASR components, each employing domain-specific clinical speech data: Chen et al. [54] applied two-stage fine-tuning on 200 h of ambulance speech (EMSSData and AISHELL-1), Xu et al. [47] used LoRA fine-tuning on 50 h of Chinese medical speech, Ilyass et al. [51] applied LoRA (r = 32) on the Darija Open Dataset (45,000+ sentences), and He et al. [59] fine-tuned the Whisper encoder with CycleGAN-augmented dysarthric speech from the UA-Speech and TORGO corpora. All four ASR training datasets were at least partially private, limiting reproducibility.

**Generative LLM Component.** The OpenAI GPT family was the most widely used LLM, appearing in 12 of the 19 studies (Figure 7b). GPT-4o was the most frequent individual model (n = 5) [46,48,49,51,52], followed by ChatGPT-3.5/GPT-3.5 (n = 6) [42,43,57,58,60,61]. The Meta LLaMA family was the second most prevalent (n = 7), particularly among studies that required model adaptation or on-device deployment [45,51,53,54,55,56,59]. Alibaba’s Qwen models appeared in four studies [47,51,54,55], while Anthropic’s Claude was used in three [50,51,57]. Baichuan models appeared in two Chinese-language studies [47,54]. One study [51] benchmarked eight LLMs across both open and closed-source families, providing a rare comparative evaluation.

Regarding model accessibility, nine studies relied exclusively on closed-source LLMs (accessed via API), six used only open-source models, and one study [51] employed both. This split has direct implications for reproducibility: studies using closed-source APIs cannot guarantee version stability or enable independent replication of results.

LLMs served as the primary component in 13 of the 19 studies, reflecting the field’s shift from transcription-centric to intelligence-centric pipelines. In the remaining six studies, LLMs played a secondary or supporting role—for example, generating error corrections on ASR output [46], producing emergency summaries from transcriptions [54], or performing machine translation [53].

**Figure 8:**
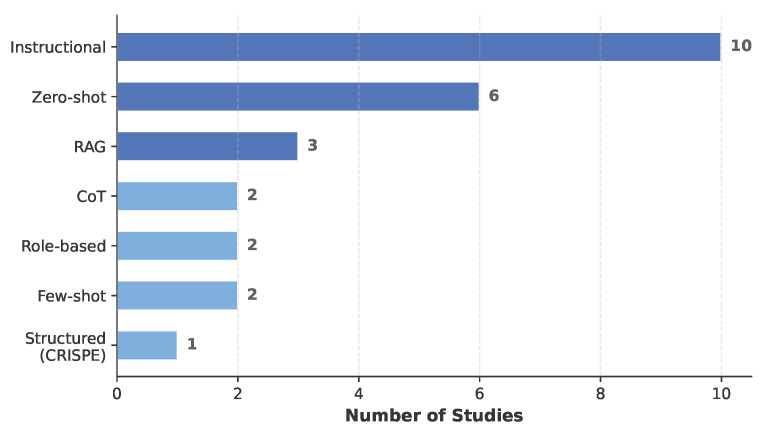
Distribution of LLM prompting techniques across the 19 included studies. Studies may employ multiple techniques; therefore, counts exceed n = 19. Instructional prompting is the most prevalent strategy, followed by zero-shot and retrieval-augmented generation (RAG).

Most studies (13 out of 19) used LLMs in their base configuration without parameter updates, relying instead on prompting strategies to steer model behavior. As shown in Figure 8, instructional prompting was the most common technique (n = 10), in which models were directed to perform specific clinical tasks through detailed task descriptions [42,43,46,47,51,52,54,57,59,61]. Zero-shot prompting was used in six studies [45,48,49,53,55,56]. Retrieval-Augmented Generation (RAG) appeared in three studies, grounding LLM outputs in clinical knowledge bases such as breast cancer guidelines [51], ophthalmic documentation corpora [47], and emergency call transcripts [50]. Chain-of-thought (CoT) reasoning was employed in two studies for Alzheimer’s detection [55] and EMR generation [60], the latter combining CoT with the CRISPE structured prompting framework. Role-based prompting, in which the LLM was assigned a clinical persona, was used in two studies for therapeutic dialogue [58] and emergency documentation [54]. Studies frequently combined multiple prompting strategies: for example, Ilyass et al. [51] combined RAG with instructional prompting, and Umer et al. [56] used both few-shot and zero-shot approaches across different model comparisons.

Only six studies applied parameter-level adaptation to their LLMs. Three used LoRA fine-tuning: Park et al. [55] fine-tuned Llama3.2-1B (r = 16) on the ADReSS dataset for Alzheimer’s classification, Xu et al. [47] adapted Qwen2-7B and Baichuan-13B (r = 8) on ophthalmic documentation corpora, and He et al. [59] fine-tuned Llama-3.1-8B (r = 8) on dysarthric speech corpora for error correction. Two studies applied full or few-shot fine-tuning: Zisquit et al. [58] fine-tuned GPT-J-7B on 3500 licensed counseling transcripts, and Umer et al. [56] used few-shot fine-tuning of LLaMA 3 on five public stress and depression benchmark datasets.

Beyond the choice of models and adaptation strategies, the tasks assigned to LLMs and PLMs reveal the functional diversity of these pipelines. Table 6 summarizes the tasks performed by generative LLMs across the 19 included studies. Clinical documentation generation was the most common task (n = 6), consistent with the administrative focus identified in RQ1. However, LLMs also served a range of other functions including classification, error correction, therapeutic dialogue, and compliance validation—reinforcing the observation that these models function as flexible reasoning components that extend well beyond transcription support.

Seven studies also employed supporting PLMs for specialized tasks alongside their generative LLM components, as summarized in Table 7. BERT and its variants were the most common, appearing in four studies primarily for classification tasks. Most PLMs were fine-tuned on domain-specific data (5 out of 7), with training datasets including psychiatric patient recordings [42], pediatric OCD therapy sessions [45], facial expression images [57], dysarthric speech corpora [59], and public stress detection benchmarks [56].

**Figure 9:**
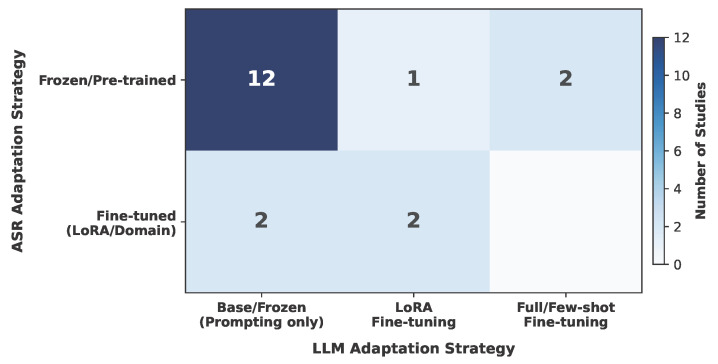
Cross-tabulation of ASR and LLM adaptation strategies (n = 19). The dominant pattern is frozen ASR with base LLMs steered through prompting only (n = 12). Only two studies fine-tuned both components.

**ASR-LLM Adaptation Patterns.** Figure 9 presents the cross-tabulation of ASR and LLM adaptation strategies across all 19 studies. The dominant pattern was frozen ASR paired with base LLMs steered through prompting alone, observed in 12 studies (63.2%). While this configuration reflects a low-barrier approach that leverages the general-purpose capabilities of pre-trained models, from a critical standpoint, this heavy reliance indicates that the field is currently prioritizing rapid proof-of-concept prototyping over rigorous clinical engineering. Relying almost exclusively on prompting without parameter-level adaptation raises significant questions about how well such systems can reliably generalize to specialized clinical vocabularies, highly noisy acoustic environments, and strict domain-specific documentation formats in sustained clinical practice.

At the opposite end of the spectrum, only two studies fine-tuned both their ASR and LLM components [47,59]. These studies addressed particularly challenging domains—ophthalmic documentation in Mandarin and dysarthric speech recognition—where off-the-shelf models proved insufficient. The remaining studies applied adaptation to one component only: two fine-tuned ASR while using base LLMs [51,54], one applied LoRA to the LLM while keeping ASR frozen [55], and two fine-tuned LLMs with frozen ASR [56,58].

Dataset availability further constrains adaptation potential. Across all components, training datasets were predominantly private or partially private: all four ASR training datasets included private data, and three of the six LLM training datasets were private or required licensing. Public datasets were more common among PLM training data (three out of five studies), partly due to the availability of established NLP benchmarks. This asymmetry suggests that the reproducibility of domain-adapted systems remains a significant challenge, particularly for ASR components trained on clinical speech data.


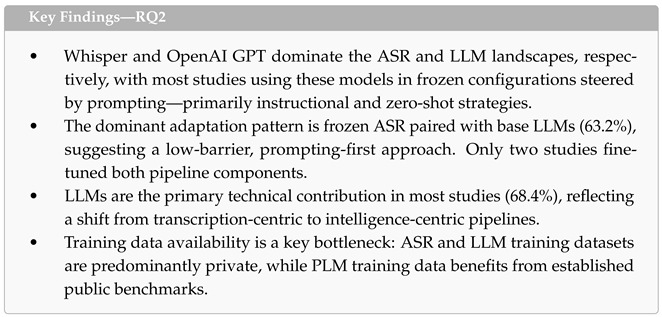


### 4.4. RQ3: Evaluation Methods

This subsection addresses the third research question: *What evaluation environment and methods, including performance metrics, are employed to assess LLM-based ASR systems in healthcare contexts?* We analyzed evaluation practices across three dimensions: the metrics used to assess ASR and LLM components, the extent of human-in-the-loop (HITL) involvement, and the methods employed for external validation beyond internal test sets.

Before presenting the metrics, it is useful to distinguish three evaluation levels that are conflated in much of the literature: (i) *ASR-level* evaluation, which measures transcription fidelity given audio input (e.g., WER, CER); (ii) *LLM-level* evaluation, which measures the quality of the generated clinical artifact given a transcript (e.g., BERTScore, human ratings, classification accuracy); and (iii) *end-to-end* evaluation, which measures the quality of the final clinical artifact given the original audio—and is therefore the only level that captures cascading errors. Of the 19 studies, 11 reported ASR-level metrics, 17 reported LLM-level metrics, and only a handful explicitly evaluated end-to-end performance with audio in and clinical artifact out [52,54,61]. The asymmetry we describe in this section therefore concerns LLM-level evaluation; end-to-end evaluation is, across all three levels, the least mature.

**Evaluation Metrics.** Eleven of the 19 studies reported ASR-specific evaluation metrics, while 17 reported LLM evaluation metrics. Table 8 summarizes the metrics identified across both components, grouped by metric family, with references to the studies employing each metric. Detailed descriptions of all reported metrics are provided in Appendix A.

ASR evaluation was dominated by word error metrics, used in 10 of the 11 studies that reported ASR-specific results. Word Error Rate (WER) was the most common metric (n = 8), followed by Character Error Rate (CER, n = 5)—the latter particularly relevant for character-based languages such as Mandarin [47,54]. One study [61] reported a comprehensive set of five word error metrics (WER, CER, MER, WIL, WIP), while another [46] introduced domain-specific variants—Dental WER (DWER) and Normalized DWER (N-DWER)—tailored to orthodontic terminology. Only one study reported a system-level ASR metric (latency) [50]. Eight studies did not report any ASR-specific evaluation, typically because ASR served a secondary role through a commercial API without modification.

LLM evaluation was more diverse, drawing on a broader range of metric families (Table 8). Human evaluation was the most prevalent approach (n = 7), encompassing manual scoring of output quality for accuracy, relevance, and formatting [54,57,60], qualitative thematic analysis [58], baseline-versus-augmented comparisons [42], ablation studies [43], chi-square testing of note quality [61], and manual error categorization [49]. Text similarity metrics were used in six studies, with BERTScore (n = 4) [46,47,49,60] and ROUGE variants (n = 5) being the most frequent, followed by BLEU (n = 2). Classification performance metrics (accuracy, F1, precision, recall) appeared in three studies focused on diagnostic tasks [45,55,56]. System-level metrics appeared in four studies and included platform adoption rates [52], RAG retrieval quality scores [51], conceptual precision [50], and hallucination rates [46].

The contrast between ASR and LLM evaluation reveals a fundamental asymmetry in evaluation maturity. ASR evaluation has converged almost entirely around word error metrics—particularly WER—providing a well-established standard that enables cross-study comparison, albeit with known limitations in capturing clinical significance. LLM evaluation, by contrast, remains fragmented across multiple metric families with no dominant standard. This fragmentation complicates cross-study comparison and makes it difficult to assess whether systems are improving over time. Furthermore, human evaluation protocols varied widely—from structured multi-dimensional scoring frameworks [60] to informal satisfaction assessments—introducing variability in evaluation rigor.

Using WER as an ASR evaluation metric for medical use under-weights clinically critical token errors, which exhibits a major limitation in the current work. A drug-name substitution, a dosage transposition, an ICD-codable diagnosis miss, or a flipped negation each carries disproportionate clinical risk but contributes the same as any other token to overall WER. Recognizing this limitation, only one study in our sample developed clinically weighted variants: Okane et al. [46] introduced Dental WER (DWER) and Normalized DWER (N-DWER), and the broader gray literature has proposed Medical Concept WER (MC-WER) as a domain-agnostic generalization [29].

**Figure 10:**
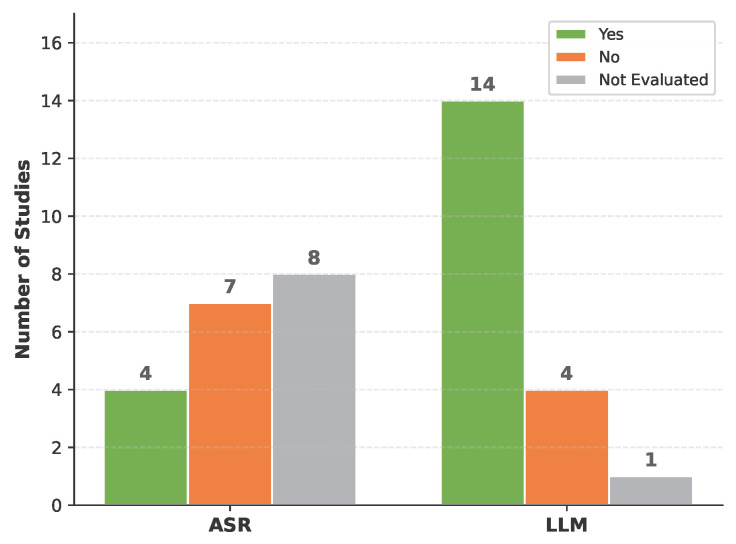
Human-in-the-loop involvement in ASR and LLM evaluation (n = 19). LLM outputs are far more likely to involve human evaluators (73.7%) than ASR transcriptions (21.1%).

**Human-in-the-Loop Evaluation.** The extent of human involvement in evaluation differed markedly between the ASR and LLM stages, as shown in Figure 10. For ASR, only four studies (21.1%) incorporated human evaluators [45,46,47,61], typically through manual transcript correction to establish gold-standard references against which automated metrics were computed. Seven studies evaluated ASR without human involvement, and eight did not evaluate ASR at all.

For LLM outputs, human involvement was substantially more common: 14 studies (73.7%) incorporated human evaluators at the LLM stage. This higher rate reflects the nature of LLM outputs—clinical notes, diagnostic summaries, and therapeutic responses—which require domain expertise to assess for accuracy, completeness, and clinical appropriateness in ways that automated metrics alone cannot capture. Forms of human involvement included clinician review and scoring of generated documentation [47,49,54,60], expert validation of compliance with clinical guidelines [51], patient and user feedback through satisfaction questionnaires [42,56,57,58], clinician-mediated quality control of system outputs before clinical use [46,52,61], and manual evaluation of translation and transcription quality [53].

**External Validation.** Twelve of the 19 studies (63.2%) conducted some form of external validation beyond internal test set evaluation, as summarized in Table 9. Clinician evaluation was the most common approach (n = 8), in which physicians, radiologists, or other domain experts assessed system outputs for accuracy, relevance, and clinical utility. Satisfaction questionnaires were used in six studies to capture user experience from clinicians, patients, or both. Clinical workflow simulation—in which the system was tested in a scenario resembling actual clinical use—appeared in four studies. Only one study achieved real-world deployment at scale: de Paula et al. [52] deployed their system with 2006 physicians in clinical practice, making it the only study to report adoption-level validation. One study conducted compliance guardrail testing for toxicity and data leakage [51].

The remaining seven studies (36.8%) did not conduct external validation [43,45,46,48,50,55,59], relying solely on automated metrics computed on internal datasets. This gap is particularly notable for diagnostic systems, where clinical validity is essential, but none of the four diagnosis-focused studies conducted external validation with end-users.

Several studies that conducted external validation also reported outcomes beyond technical accuracy, including documentation time reduction [47,54,57,61], user satisfaction [42,52,56,58], and cost-effectiveness [49]. These practical outcomes provide evidence of clinical utility that automated metrics alone cannot capture, yet they were not systematically reported across all studies, limiting the ability to compare real-world impact.


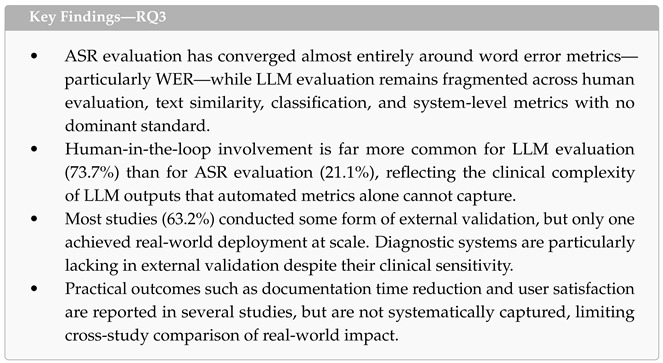


### 4.5. RQ4: Reported Insights and Challenges

This subsection addresses the fourth research question: *What are the benefits, implementation challenges, and ethical considerations that have been reported in the included studies?* We synthesize findings across three dimensions: reported clinical benefits, implementation challenges, and ethical considerations, including privacy, equity, and reproducibility.

**Reported Clinical Benefits.** The most frequently reported benefit was a reduction in documentation time, cited in seven studies. The magnitude of time savings varied considerably: Gasque et al. [61] reported the most dramatic reduction, from approximately 16 min to 1 min per consultation note; Xu et al. [47] reported a 62% reduction in documentation time; Wu et al. [57] observed report writing time reduced by over 50% with a 70% decrease in administrative workload; and Chen et al. [54] reported a reduction from 20 to 14 min for emergency documentation. Ding et al. [60] noted reduced consultation time for junior clinicians, and Busch et al. [49] assessed cost-effectiveness alongside correction time. User satisfaction was reported in five studies [42,52,56,57,58], with de Paula et al. [52] providing the largest-scale evidence through deployment with 2006 physicians who reported high satisfaction and reduced documentation burden. Beyond efficiency gains, two therapy-oriented studies reported qualitative benefits: improved patient self-reflection [58] and 75% of psychiatric patients finding therapeutic exercises helpful [42].

**Implementation Challenges.** The challenges reported across the included studies clustered into four categories. First, *acoustic and environmental robustness* was a recurring concern. Chen et al. [54] specifically addressed the challenge of in-ambulance noise through a custom noise-robust ASR model, while several studies acknowledged performance degradation in noisy or multi-speaker clinical environments [53,61]. Second, *language and dialect limitations* were widely acknowledged. Five studies explicitly noted that their systems were limited to a single language [42,52,54,57,58], and six studies identified accent and dialect variation as a source of reduced accuracy [46,48,51,52,54,61]. Third, *domain-specific vocabulary* posed challenges for off-the-shelf models, motivating the domain-specific fine-tuning efforts described in RQ2—particularly for orthodontic terminology [46], ophthalmic documentation [47], and Darija-French code-switching in oncology [51]. Fourth, *model reliability concerns* were noted in several studies. Hallucination—the generation of clinically unsupported content—was explicitly measured in one study [46] and implicitly addressed through mandatory clinician review workflows in others [52,54,61]. The reliance on closed-source APIs (9 out of 19 studies) introduced additional challenges related to version stability, latency dependence, and lack of control over model behavior.

**Figure 11:**
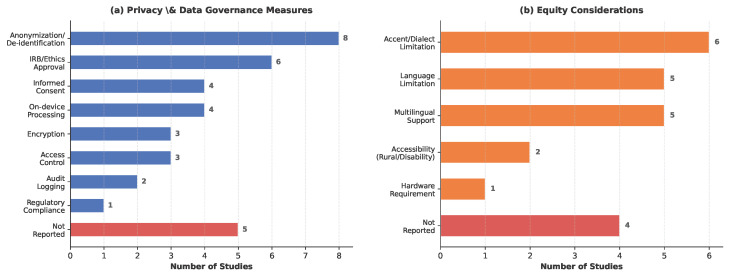
(**a**) Privacy and data governance measures and (**b**) equity considerations reported across the 19 included studies. Studies may report multiple measures; therefore, counts may exceed n = 19. Five studies did not report any privacy measures, and four did not address equity considerations.

**Privacy and Data Governance.** Figure 11a summarizes the privacy and data governance measures reported across the 19 studies. Anonymization or de-identification of patient data was the most commonly reported measure (n = 8), followed by IRB or ethics committee approval (n = 6) and informed consent procedures (n = 4). Four studies adopted on-device processing architectures to avoid transmitting clinical speech data to external servers [47,53,55,56]. Technical safeguards including encryption (n = 3) [50,52,57], access control mechanisms (n = 3) [50,51,54], and audit logging (n = 2) [54,57] were reported in a smaller number of studies. One study [50] implemented a blockchain-based architecture using Arweave decentralized storage and Solana multi-signature access control for emergency call archiving. Only one study explicitly reported compliance with a regional data protection regulation (Brazilian LGPD) [52]. Notably, five studies (26.3%) did not report any privacy or data governance measures [43,45,46,48,59], representing a significant reporting gap given the sensitivity of clinical speech data.

**Equity Considerations.** Figure 11b presents the equity-related issues reported across the included studies. Accent and dialect variation was the most frequently acknowledged concern (n = 6), with studies noting performance degradation for speakers with non-standard accents, regional dialects, or code-switching patterns [46,48,51,52,54,61]. Five studies explicitly acknowledged language limitations—that their systems were designed for and tested in a single language, limiting generalizability to other linguistic populations. Five studies addressed multilingual support or low-resource language challenges as part of their design or discussion [48,49,50,53,56]. Two studies addressed accessibility for underserved populations: Wu et al. [57] designed their teletherapy platform for rural communities while acknowledging digital exclusion risks, and He et al. [59] specifically targeted dysarthric speakers whose speech impairments render standard ASR systems ineffective. One study noted hardware accessibility as a barrier, requiring VR equipment for its counseling platform [58]. Four studies (21.1%) did not report any equity considerations [43,45,47,55]. It bears emphasis that 15 studies acknowledged at least one equity consideration (most commonly accent, dialect, or language coverage), whereas none performed a planned, pre-registered subgroup test for fairness with statistical comparison across demographic subgroups. We therefore distinguish between equity acknowledgment and equity evaluation: the former is widespread but the latter is absent across this sample.

**Reproducibility.** Only five of the 19 studies (26.3%) provided a replication package with publicly available code [45,47,50,55,60]. The remaining 14 studies did not share code, models, or data processing pipelines. When combined with the prevalence of private training datasets (as noted in RQ2) and the reliance on closed-source LLM APIs (9 out of 19 studies), the overall reproducibility landscape is limited. This is particularly concerning for studies proposing novel clinical systems, where independent verification of reported results is essential for clinical adoption and regulatory approval.


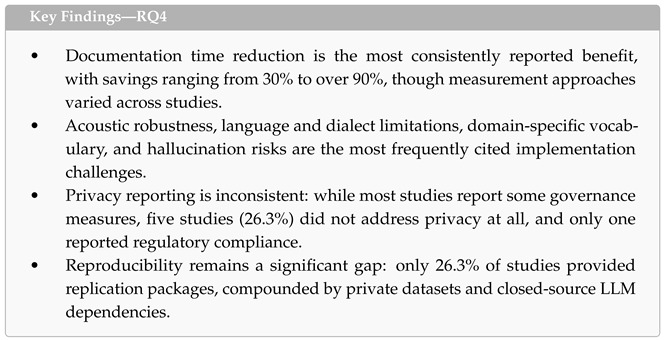


## 5. Discussion

### 5.1. Insights and Practical Implications

Based on the synthesis of findings across the four research questions, we highlight the following insights and practical implications for researchers and practitioners working with LLM-based ASR systems in healthcare.

1. **LLMs serve as the primary component in most pipelines, not ASR.** In 13 of the 19 included studies, the LLM held the primary role while ASR served as a secondary transcription input. This distribution suggests that the research community increasingly views these systems as clinical intelligence pipelines in which speech recognition is an input mechanism rather than the core contribution. Practitioners designing such systems should allocate evaluation and adaptation effort accordingly, with particular attention to the LLM component’s capacity for clinical reasoning, summarization, and structured output generation.
2. **Off-the-shelf deployment is common but may not be sufficient for all contexts.** Fifteen of the 19 studies used ASR models without any domain-specific adaptation, and 13 used LLMs in their base configuration with prompting alone. While this low-barrier approach enables rapid prototyping, the four studies that did fine-tune their ASR components did so because general-purpose models proved inadequate for underserved languages [51], atypical speech patterns [59], noisy clinical environments [54], or specialized medical terminology [47]. Organizations considering deployment should evaluate off-the-shelf model performance on their specific clinical populations and acoustic conditions before relying on frozen configurations.
3. **LLM applications extend well beyond clinical documentation.** Although administrative documentation was the most common application context (n = 7), LLMs also performed ASR error correction [46,59], clinical screening and classification [55,56], therapeutic dialogue generation [42,58], synthetic training data generation [42], regulatory compliance checking [51], and semantic speech reconstruction [50]. This breadth suggests that the integration of LLMs with ASR has potential across a wider range of healthcare workflows than documentation alone, though each application introduces distinct evaluation and safety requirements. This pattern extends to subscription-based literature; for instance, VoxRad demonstrated template-guided ASR-LLM radiology reporting with HIPAA-compliant local hosting [62], and stroke rehabilitation models achieved BLEU scores of 30.2 in multimodal audio-video speech tasks [63].
4. **Early evidence suggests multimodal LLMs may offer a pathway to unify the ASR-LLM pipeline.** GPT-4o was used as both the ASR and LLM component in two studies [46,49], bypassing the traditional two-stage architecture. This approach simplifies deployment and reduces integration complexity, but it also concentrates dependency on a single proprietary model, raising concerns about version stability, auditability, and regulatory compliance that merit further investigation.
5. **On-device processing is emerging as a privacy-preserving deployment strategy.** Four studies deployed models locally to avoid transmitting patient audio to external servers [47,53,55,56]. This approach is relevant in jurisdictions with strict data sovereignty requirements and may help address clinician concerns about patient data being processed by third-party cloud services. However, on-device deployment imposes constraints on model size and computational resources, creating a trade-off between privacy and capability that warrants further study. Recent work corroborates the feasibility of local deployment; VoxRad achieved high accuracy in radiology reporting while maintaining HIPAA compliance through locally hosted ASR and LLM processing [62].
6. **Reproducibility remains limited.** Only five of the 19 studies provided a replication package with publicly available code or models. The reliance on closed-source commercial APIs (nine of 19 studies) introduces a critical methodological vulnerability for clinical translation: the complete loss of version control. Because proprietary models are routinely and silently updated by their vendors, a clinical pipeline rigorously validated on a specific API version today may exhibit entirely altered behaviors tomorrow—such as shifting hallucination rates, modified clinical reasoning, or different output formatting. This lack of transparency fundamentally breaks traditional clinical change-management protocols, as researchers and health systems cannot lock the model’s state, audit the underlying parameter updates, or guarantee longitudinal verifiability. Addressing this gap requires a concerted shift toward locally hostable, open-source clinical ASR-LLM pipelines and the development of shared healthcare-specific benchmark datasets to ensure safe and reproducible clinical deployment.
7. **Equity concerns are acknowledged but rarely evaluated systematically.** Fifteen of the 19 studies mentioned at least one equity consideration—such as multilingual support, accent sensitivity, or accessibility for speech-impaired populations—yet none of the included studies made systematic bias evaluation or fairness testing a primary contribution. Moving from acknowledgment to rigorous evaluation will require dedicated benchmarks that capture the diversity of clinical speech, including regional accents, code-switching patterns, and atypical speech.
8. **Human oversight is widely practiced but inconsistently reported.** Fifteen of the 19 studies incorporated some form of human-in-the-loop mechanism, ranging from clinician review of generated notes to manual correction of ASR output to patient satisfaction surveys. However, the nature, rigor, and scope of human oversight varied considerably across studies. Standardized reporting of human oversight mechanisms—including who reviewed the output, what criteria were applied, and whether human corrections were fed back into the system—would improve comparability and support the development of clinical governance frameworks for these systems.
9. **Evaluation practice focuses on technical accuracy rather than clinical validity.** The current evaluation landscape—in which aggregate WER is the dominant ASR metric and human ratings are reported without inter-rater reliability—is insufficient for clinical deployment decisions. We propose two minimum requirements for future studies. First, at least one domain-weighted error metric should accompany aggregate WER: clinically critical tokens such as drug names, dosages, diagnoses, and negations carry disproportionate risk and should be tracked separately, as demonstrated by the DWER and N-DWER variants introduced in our sample [46] and the broader proposal of Medical Concept WER [29]. Second, any study that uses human raters to assess LLM output quality must report inter-rater reliability (ICC, weighted Cohen’s κ, or Krippendorff’s α); without it, human evaluation scores cannot be meaningfully compared across studies or used as a basis for clinical adoption decisions. These two requirements are low-cost, well-established in adjacent fields, and their absence across all 19 included studies represents the single most actionable reporting gap identified by this review.

### 5.2. Clinical Safety and Regulatory Considerations

The results reported in our synthesis reveal a consistent pattern: the included studies are predominantly designed and evaluated as research prototypes rather than regulated clinical devices. A critical, underexplored risk in these multi-component pipelines is the phenomenon of *cascading errors*—in which minor ASR misrecognitions are hallucinogenically amplified by a downstream LLM into authoritative-sounding, yet factually incorrect, clinical narratives. Mitigating this risk requires evidence-based human-in-the-loop (HITL) best practices. Safe deployment mandates that these systems remain strictly assistive; they must feature mandatory, auditable clinician review workflows where outputs are verified and signed off before entering the Electronic Health Record (EHR).

Furthermore, navigating the regulatory landscape is essential for clinical translation. Current literature lacks comprehensive analysis of frameworks such as the FDA’s Software as a Medical Device (SaMD) guidelines or European CE marking requirements, which heavily scrutinize the boundary between assistive documentation and autonomous clinical decision support. Additionally, the prevalent reliance on proprietary, cloud-based LLM APIs introduces severe data governance vulnerabilities. Transmitting sensitive patient audio or raw transcripts to third-party servers necessitates stringent compliance with HIPAA and GDPR, demanding robust de-identification pipelines and zero-data-retention agreements that are rarely detailed in the current literature.

Taken together, these findings point to a translational gap: while promising accuracy and efficiency results have been reported, the evidence base required for safe clinical deployment—end-to-end error characterization, formal regulatory positioning, and systematic human-oversight reporting—has not yet been established. Closing this gap is a prerequisite for moving from research-prototype status to regulated-device deployment, and it must be a primary focus of the next generation of ASR–LLM healthcare studies.

### 5.3. Toward a Standardized Evaluation Framework for Clinical ASR–LLM Systems

Synthesizing the gaps identified in Section 4.4 and Section 4.5, we propose a four-layer evaluation framework as a starting point for standardization. This framework is offered as a recommendation grounded in the present evidence, not as a finished standard.

**Layer 1—ASR fidelity.** Report aggregate WER (and CER for character-based languages) *together with* a clinically weighted variant such as Medical Concept WER  [29] or a domain-tailored equivalent (DWER, N-DWER). Report subgroup-stratified results by accent, dialect, and any patient demographic for which audio is available, in the spirit of the disparities documented by Zolnoori et al. [27] and DiChristofano et al. [28].

**Layer 2—LLM faithfulness.** Report hallucination and omission rates with explicit denominators (per-note, per-token, or per-clinical-claim, as appropriate). Where automated text-similarity metrics (BERTScore, ROUGE) are reported, clarify what they measure, and pair them with at least one clinical correctness metric.

**Layer 3—End-to-end clinical safety.** As established in our evaluation taxonomy (Section 4.4), end-to-end evaluation—measuring system performance directly from audio to clinical artifact—is essential for deployment decisions, as it captures cascading errors that component-level metrics miss. Evaluate the full audio-to-artifact pipeline, not only the transcript or only the LLM output. Include cascading-error analysis (Section 5.2) and a harm-grading rubric for errors that survive automated checks.

**Layer 4—Reporting rigor for human evaluation.** For every study that uses human raters, disclose: panel size and composition (e.g., specialist clinicians vs. medical students); blinding to system identity; rubric instrument; and inter-rater reliability statistics (weighted Cohen’s κ, ICC, or Krippendorff’s α, depending on rating type).

**Reproducibility requirements.** To address the vulnerabilities introduced by closed-source APIs, future studies must disclose: (a) exact API versions and timestamps for all model calls (e.g., gpt-4-turbo-2024-04-09); (b) complete prompt strings and system messages in supplementary materials or model cards; (c) versioned snapshots or containerized environments to enable future replication; and (d) where feasible, parallel evaluation on open-source alternatives to demonstrate generalizability beyond proprietary systems. For multi-institutional validation, federated evaluation frameworks that preserve data privacy while enabling cross-site reproducibility should be prioritized.

**Fairness checklist.** As an actionable component of Layers 1–3, future studies should report: (a) the demographic composition of training and evaluation data; (b) subgroup-stratified WER and clinically weighted error rates; (c) at least one “unseen-population” hold-out (e.g., a regional accent not present in training) to expose generalization gaps; and (d) explicit thresholds for what counts as a substantive disparity.

**Figure 12:**
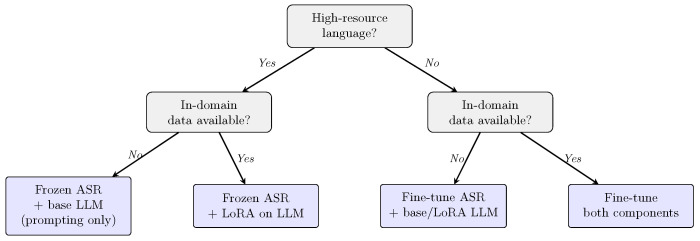
Deployment decision tree. Branching is by (i) language resource level (high vs. low), (ii) availability of in-domain training data, (iii) computational/budget constraints, and (iv) whether on-device inference is required for privacy reasons. Terminal configurations align with the four observed adaptation patterns in Section 4.3.

**Deployment decision tree.** Finally, in Figure 12 we summarize a decision tree to help institutions choose between frozen-prompt, LoRA-on-LLM, fine-tune-ASR-only, and fine-tune-both configurations, conditioned on language resource level, in-domain data availability, computational constraints, and on-device requirements.

### 5.4. Limitations

This review has several limitations:

1. The inclusion criteria required both ASR and LLM components, excluding studies that advanced either technology in isolation for healthcare applications. This design choice was deliberate, as the review specifically targets the intersection of these technologies to understand how they function together in clinical pipelines. Future reviews could examine standalone ASR or standalone LLM healthcare applications to complement the findings presented here.
2. The publication window (2022–2025) resulted in a temporally skewed sample, with 17 of 19 studies from 2025, reflecting the recency of the field. This concentration is expected, given that the widespread integration of LLMs with ASR in healthcare began only after the release of models such as ChatGPT and Whisper in late 2022. As more studies emerge, future reviews should revisit these findings to assess whether the patterns identified here—such as the dominance of frozen configurations and the scarcity of empirical evaluations—persist or evolve.
3. As a scoping review, this study prioritized breadth of landscape mapping over formal quality assessment of individual studies. While this approach aligns with PRISMA-ScR methodology [19,41], it limits our ability to differentiate robust from weaker evidence or to weight findings by study quality. Future systematic reviews addressing specific clinical questions would benefit from formal quality appraisal using validated tools such as MI-CLAIM [64] for clinical AI studies or PROBAST [65] for prediction model assessment, enabling evidence grading and clinical recommendation development.
4. The rapid pace of ASR and LLM development means that newer models, techniques, and clinical deployments may have emerged since the search was conducted. This is an inherent challenge in reviewing any fast-moving technology area. To partially address this, the search window was extended through December 2025 to capture the most recent available work. Periodic updates to this review would help track the trajectory of the field.
5. Restricting inclusion to open-access, peer-reviewed publications excludes two categories of evidence: subscription-journal studies and gray literature (preprints, technical reports). The subscription-access constraint may under-represent high-quality clinical deployment studies and commercial system evaluations often published in related journals. The gray literature exclusion means recent preprints and technical reports are not captured, potentially missing developments too recent for traditional peer review. These were deliberate methodological choices to ensure reproducibility and peer-review fidelity [19,41]. Gray literature, while valuable for timeliness, lacks the quality assurance mechanisms of peer review that are essential for systematic evidence synthesis intended to inform clinical deployment [64]. Our findings thus reflect the peer-reviewed, open-access literature rather than the complete development landscape. Representative subscription-based studies corroborate key findings on documentation applications [62], multimodal integration [63], and clinical performance benchmarks—a VR-based triage system achieved 95.1% task success with 4.61/5 naturalness ratings [66].

## 6. Conclusions

This scoping review examined 19 studies published between 2023 and 2025 to map the emerging landscape of LLM-based ASR systems in healthcare. This review synthesizes the peer-reviewed, open-access published literature on LLM-based ASR in healthcare; subscription-based and gray literature sources fall outside our scope. The findings, organized around four research questions, reveal a field that is growing rapidly but remains in its early stages of maturity.

In terms of clinical applications (RQ1), administrative documentation and diagnosis are the most common use cases, while emergency and homecare settings remain underexplored. A notable pattern emerged in the relationship between language and deployment: non-English studies tend to target clinical environments for workflow-driven tasks, whereas English-only studies concentrate on diagnostic applications in telehealth and experimental settings.

Regarding technical architectures (RQ2), the reviewed literature heavily favored Whisper for ASR and OpenAI GPT models for LLM processing, with the majority of studies using both components in frozen configurations steered through prompting—primarily instructional and zero-shot strategies. Only two studies fine-tuned both their ASR and LLM components, and training datasets remain predominantly private, limiting reproducibility. The emergence of multimodal models such as GPT-4o serving dual ASR-LLM roles signals a potential shift toward unified pipelines, though this raises new questions around auditability and vendor dependency.

Evaluation practices (RQ3) revealed a clear asymmetry: ASR evaluation within the reviewed studies predominantly relied on word error rate as a standard metric, while LLM evaluation remains fragmented across human evaluation, text similarity, classification, and system-level metrics with no established standard. Human-in-the-loop involvement was far more common for LLM outputs (73.7%) than for ASR (21.1%), and although most studies conducted some form of external validation, only one achieved real-world deployment at scale.

The reported insights and challenges (RQ4) highlighted documentation time reduction as the most consistently reported benefit, with savings ranging from 30% to over 90%. However, privacy reporting was inconsistent across studies, equity concerns were acknowledged but rarely evaluated systematically, and only five studies provided replication packages.

Overall, these findings point to several critical directions for future work:

1. **Standardized evaluation frameworks for clinical LLM outputs.** The field urgently needs consensus metrics including hallucination and omission rates with explicit denominators, harm-grading rubrics for cascading errors, and mandatory reporting of inter-rater reliability (ICC, Cohen’s κ, or Krippendorff’s α) for human evaluation panels. Without these standards, cross-study comparison remains impossible and clinical adoption lacks an evidence base.
2. **Shared multilingual clinical speech benchmarks.** Standardized datasets with subgroup-stratified test sets and clinically weighted error metrics (e.g., MC-WER, DWER) would expose the performance disparities that aggregate WER conceals. These benchmarks should span multiple languages, accents, and clinical contexts to enable systematic fairness evaluation.
3. **Systematic bias and fairness testing.** The field must move from widespread equity acknowledgment (15 of 19 studies) to empirical evaluation (currently absent). Future work should mandate pre-registered subgroup analyses with statistical comparison across demographic groups, unseen-population hold-outs, and explicit disparity thresholds.
4. **Open-source pipelines and reproducible research infrastructure.** Progress on all fronts depends on commitments to open-source models, public datasets, replication packages, and federated evaluation frameworks that overcome the reproducibility barriers imposed by closed-source APIs (9 of 19 studies) and private clinical-speech corpora.

As the integration of ASR and LLM technologies in healthcare continues to accelerate, rigorous empirical evaluation in real clinical environments will be essential to ensure that these systems deliver on their potential to reduce clinician burden, improve documentation quality, and expand access to care.

## Figures and Tables

**Table 1:** Comparison of this scoping review with related recent reviews on speech and language technologies in healthcare.

Review	Year	PRISMA	Healthcare Domain	ASR Focus	LLM Focus	ASR-LLM Integration	Evaluation Analysis	Ethics and Challenges
Van Buchem et al. [35]	2021	✕	Digital scribes	✓	✕	✕	✓	✕
Zhang et al. [1]	2023	✕	Smart hospitals	✓	✕	✕	✕	✕
Jordan et al. [36]	2025	✓	Mental health (SER *)	✓	✕	✕	✓	✕
Ng et al. [2]	2025	✓	Clinical documentation	✓	✕	✕	✓	✕
Sasseville et al. [37]	2025	✓	AI scribes	✓	✕	✕	✓	✕
**This Review**	**2025**	✓	**Comprehensive (all domains)**	✓	✓	✓	✓	✓

* SER = Speech Emotion Recognition (analyzes emotional states from voice, not transcription). **Legend:** ✓ = addressed; ✕ = not addressed or limited coverage. **Column Definitions: ASR Focus**: Automatic Speech Recognition (speech-to-text transcription) as a primary topic. **LLM Focus**: Large Language Models as a primary topic. **ASR-LLM Integration**: Explicit focus on combining ASR with LLMs for healthcare applications. **Evaluation Analysis**: Systematic analysis of evaluation methods, metrics, and benchmarks. **Ethics and Challenges**: Discussion of ethical considerations, implementation challenges, and limitations.

**Table 2:** Positioning of this review relative to recent general-domain ASR-LLM surveys.

Reference	Their Contribution	What This Review Adds
Muthusamy et al. (2025) [38]	60-year historical overview; ASR evolution from HMM to LLMs	Application taxonomy (RQ1); systematic evaluation critique (RQ3); reproducibility quantification (5/19 packages)
Yang et al. (2025) [39]	Technical fusion architectures; modality alignment methods	Architectural adaptation patterns in practice (RQ2); deployment challenge synthesis (RQ4); clinical validity framework
Cui et al. (2025) [40]	Speech LM advances; training paradigms; benchmark datasets	Real-world application mapping; evaluation fragmentation analysis; translational gap identification

**Table 4:** Application contexts identified across the 19 included studies.

Application Context	Description
Administrative	Clinical documentation tasks such as generating medical notes, structuring EMRs, transcribing radiology reports, and summarizing consultations.
Diagnosis	Systems that analyze speech to support clinical decision-making, including screening for cognitive decline, detecting stress, and automated coding of therapy sessions.
Therapy	Applications that support therapeutic processes, including dialogue systems for counseling, teletherapy augmentation, VR-based counseling, and bilingual patient education.
Doctor–patient communication	Tools that facilitate or improve spoken interaction between clinicians and patients, including speech reconstruction for degraded signals, EMR generation from consultations, and speech recognition for atypical speech.

**Table 5:** Intended application settings identified across the 19 included studies.

Application Setting	Description
Clinical	Outpatient clinics, primary care facilities, and specialty practices where patients receive scheduled consultations or therapy sessions.
Hospital	Inpatient or departmental settings within hospitals, including radiology, ophthalmology, and orthodontics units.
Telehealth	Remote care delivered through digital platforms, including video-based therapy, VR-based counseling, and remote screening tools.
Homecare	Patient-initiated use in home environments, such as smartphone-based monitoring, self-administered assessments, and web-based health education.
Emergency	Prehospital and acute settings, including ambulance-based documentation and emergency communication systems.

**Table 6:** Tasks performed by generative LLMs across the 19 included studies.

Task	Description	Studies
Clinical documentation	Generating clinical notes, EMRs, medical records, or radiology reports from speech input.	[47,49,52,57,60,61]
Classification/screening	Classifying speech content for diagnostic purposes, including AD detection and stress detection.	[45,55,56]
Emergency/diagnostic summary	Generating structured summaries from prehospital or clinical speech for urgent decision support.	[51,54]
ASR error correction	Post-processing ASR transcriptions to correct domain-specific recognition errors.	[46,59]
Therapeutic dialogue	Generating counselor-style responses in therapeutic or mental health interactions.	[42,58]
Synthetic data generation	Producing artificial training data to augment limited clinical datasets.	[42]
Compliance validation	Checking clinical outputs against guideline-based rules for regulatory adherence.	[51]
Speech reconstruction	Semantically reconstructing degraded or incomplete speech signals.	[50]
Patient education (QA)	Answering patient questions and generating educational content.	[48]
Machine translation	Translating clinical speech across languages.	[53]
Fluency/opinion evaluation	Assessing speech fluency and generating clinical opinions from transcripts.	[43]

**Table 7:** Tasks performed by supporting PLMs across the 19 included studies.

Task	Description	Studies
Text classification	Intent recognition, emotion detection, sentiment analysis, and stress classification.	[42,45,56]
Feature extraction	Extracting acoustic features (Wav2Vec 2.0) or textual embeddings (BERT) from speech or transcripts.	[43]
Semantic retrieval	Matching patient queries to relevant educational content using embedding similarity.	[48]
Data augmentation	Generating augmented dysarthric speech samples via adversarial training (CycleGAN).	[59]
Facial expression analysis	Classifying patient facial expressions during teletherapy sessions (Swin Transformer).	[57]

**Table 8:** Evaluation metrics identified across the 19 included studies, grouped by metric family and pipeline component. Frequency indicates the number of studies using each metric.

Metric Family	Comp.	Metric	Freq.	Studies
Word Error	ASR	WER	8	[42,45,46,47,51,55,59,61]
	ASR	CER	5	[46,51,54,57,61]
	ASR	DWER; N-DWER; uWER	1 ea.	[46]
	ASR	MER; WIL; WIP	1 ea.	[61]
	ASR	SRR; RAR	1 ea.	[42,57]
Text Similarity	LLM	BERTScore	4	[46,47,49,60]
	LLM	ROUGE (variants)	5	[47,49,50,53,60]
	LLM	BLEU	2	[47,50]
	LLM	BARTScore	1	[46]
Classification	LLM	Accuracy	4	[45,54,55,56]
	LLM	F1; precision; recall	3 ea.	[53,55,56]
	LLM	AUC	1	[45]
Human Evaluation	LLM	Manual quality scoring	3	[54,57,60]
	LLM	Qualitative thematic analysis	1	[58]
	LLM	Baseline vs. augmented	1	[42]
	LLM	Ablation study	1	[43]
	LLM	Chi-square test	1	[61]
	LLM	Manual error categorization	1	[49]
System-Level	ASR	Latency	1	[50]
	LLM	Hallucination rate	1	[46]
	LLM	Platform usage; activation rate	1 ea.	[52]
	LLM	RAG Triad; conceptual prec.	1 ea.	[50,51]

**Table 9:** External validation methods employed across the 19 included studies. Studies may use multiple methods.

Method	Description	Freq.	Studies
Clinician evaluation	Domain experts assess outputs for accuracy, relevance, and clinical utility.	8	[47,49,51,52,53,54,57,60]
Satisfaction questionnaire	Structured survey capturing user experience from clinicians, patients, or both.	6	[42,47,52,56,57,58]
Workflow simulation	System tested in a scenario resembling real clinical use.	4	[49,53,54,57]
Real-world deployment	System deployed in routine clinical practice at scale.	1	[52]
Compliance testing	Guardrail testing for toxicity, data leakage, and guideline adherence.	1	[51]
Not applied		7	[43,45,46,48,50,55,59]

## Data Availability

No new data were created or analyzed in this study. This is a scoping review based entirely on previously published, open-access literature. The data extraction form and full list of included studies are available within the article.

## Appendix A. Evaluation Metric Descriptions

Table A1, Table A2, Table A3, Table A4 and Table A5 provide descriptions of the evaluation metrics identified across the 19 included studies, organized by metric family.

**Table A1:** Word error metrics used for ASR evaluation.

Metric	Description
WER	Word Error Rate. Proportion of words incorrectly inserted, deleted, or substituted relative to a reference transcript.
CER	Character Error Rate. Character-level analog of WER; particularly relevant for character-based languages such as Mandarin.
DWER	Dental Word Error Rate. Domain-specific variant that weights errors on clinical terminology more heavily.
N-DWER	Normalized DWER. Length-normalized version of DWER for cross-transcript comparison.
uWER	Unweighted WER. Treats all word types equally without frequency weighting.
MER	Match Error Rate. Proportion of matched word pairs containing errors, accounting for alignment.
WIL	Word Information Lost. Proportion of word-level information lost between reference and hypothesis.
WIP	Word Information Preserved. Complement of WIL; proportion of information correctly retained.
SRR	Sentence Recognition Rate. Proportion of sentences transcribed entirely without error.
RAR	Recognition Accuracy Rate. Proportion of correctly recognized speech units.

**Table A2:** Text similarity metrics used for ASR and LLM evaluation.

Metric	Description
BERTScore	Semantic similarity between generated and reference texts using contextual BERT embeddings, capturing meaning beyond surface-level overlap.
ROUGE	Recall-Oriented Understudy for Gisting Evaluation. N-gram overlap between generated and reference texts (ROUGE-1, ROUGE-2, ROUGE-L).
BLEU	Bilingual Evaluation Understudy. Precision of n-gram overlap, originally developed for machine translation.
BARTScore	Log-likelihood of generated text given the reference using a pre-trained BART model.

**Table A3:** Classification performance metrics used for LLM and PLM evaluation.

Metric	Description
Accuracy	Proportion of correctly classified instances out of all instances.
Precision	Proportion of true positives among all positive predictions.
Recall	Proportion of true positive instances correctly identified out of all actual positives.
F1-score	Harmonic mean of precision and recall.
AUC	Area Under the ROC Curve. Measures discrimination ability across all classification thresholds.

**Table A4:** Human evaluation methods used for LLM evaluation.

Method	Description
Manual quality scoring	Domain experts rate outputs on predefined dimensions (e.g., accuracy, relevance, completeness).
Thematic analysis	Systematic identification of themes in user feedback through qualitative coding.
Baseline vs. augmented	Performance comparison with and without a specific component (e.g., LLM-augmented data).
Ablation study	Systematic removal of model components to quantify individual contributions.
Chi-square test	Statistical comparison of observed versus expected frequencies in categorical outcomes.
Error categorization	Reviewers identify, classify, and rate the severity of errors in outputs.

**Table A5:** System-level metrics used for ASR and LLM evaluation.

Metric	Description
Latency	Time between speech input and system output; critical for real-time applications.
Hallucination Rate	Proportion of generated content unsupported by or contradicting the source input.
Platform usage	Adoption metrics tracking active users and frequency of system use in deployment.
RAG Triad score	Composite score for RAG quality across context relevance, groundedness, and answer relevance.
Conceptual Precision	Proportion of semantically meaningful concepts correctly preserved from input to output.

## Appendix B. Database Search Queries

The exact queries used in each of the four databases on the date of the final search 31 December 2025) are reproduced below. All queries combine three concept blocks—ASR terminology, healthcare domain terms, and LLM identifiers—using the AND operator. Date filters were applied at the database UI level.

### Appendix B.1. PubMed

("automatic speech recognition" OR "speech recognition" OR

 "speech-to-text" OR "voice recognition")

AND

("healthcare" OR "medicine" OR "clinical" OR "patient" OR

 "hospital" OR "medical")

AND

("large language model" OR "large language models" OR "LLM" OR

 "LLMs" OR "GPT" OR "Whisper")

AND

("2022/01/01"[Date - Publication] : "2025/12/31"[Date - Publication])

### Appendix B.2. Scopus

TITLE-ABS-KEY(

  ("automatic speech recognition" OR "speech recognition" OR

   "speech-to-text" OR "voice recognition")

  AND

  ("healthcare" OR "medicine" OR "clinical" OR "patient" OR

   "hospital" OR "medical")

  AND

  ("large language model" OR "LLM" OR "LLMs" OR "GPT" OR "Whisper")

)

AND PUBYEAR > 2021 AND PUBYEAR < 2026

AND DOCTYPE(ar) AND OPENACCESS(1)

### Appendix B.3. IEEE Xplore

("All Metadata":"automatic speech recognition" OR

 "All Metadata":"speech recognition" OR

 "All Metadata":"speech-to-text" OR

 "All Metadata":"voice recognition")

AND

("All Metadata":"healthcare" OR "All Metadata":"medicine" OR

 "All Metadata":"clinical" OR "All Metadata":"patient" OR

 "All Metadata":"hospital" OR "All Metadata":"medical")

AND

("All Metadata":"large language model" OR "All Metadata":"LLM" OR

 "All Metadata":"GPT" OR "All Metadata":"Whisper")

Filters applied: 2022--2025; Open Access only.

### Appendix B.4. Web of Science

TS = (

  ("automatic speech recognition" OR "speech recognition" OR

   "speech-to-text" OR "voice recognition")

  AND

  ("healthcare" OR "medicine" OR "clinical" OR "patient" OR

   "hospital" OR "medical")

  AND

  ("large language model" OR "LLM" OR "LLMs" OR "GPT" OR "Whisper")

)

Refined by: PY=(2022 OR 2023 OR 2024 OR 2025);

Document Types: ARTICLE; Open Access only.

### Appendix B.5. Validation Set

The validation set used to verify the sensitivity of the search strings consisted of five studies spanning the four anticipated application contexts, with at least one non-English study: (administrative documentation, multilingual deployment) [52]; (diagnosis, English) [43]; (therapy, English) [58]; (clinical documentation in Spanish) [61]; and (diagnosis with on-device LLM) [55]. The final query was required to retrieve all five validation studies before being accepted.
